# Delayed diagnosis of retroperitoneal duodenal perforation after endoscopic treatment: Management challenges and a novel surgical strategy case series reports

**DOI:** 10.1016/j.ijscr.2025.111637

**Published:** 2025-07-09

**Authors:** Yuan Liu, Jianning Song, Jun Li, Xiujing Sun, Jiugang Song, Hongwei Yao

**Affiliations:** a Department of General Surgery, Beijing Friendship Hospital, Capital Medical University, National Clinical Research Center for Digestive Diseases, Beijing, China; b Department of Gastroenterology, Beijing Friendship Hospital, Capital Medical University, National Clinical Research Center for Digestive Disease, Beijing, China; c Department of Clincal Laboratory, Beijing Friendship Hospital, Capital Medical Univercity, Beijing, China

**Keywords:** Endoscopic, Duodenum perforation, Delayed diagnosis, Case series, General surgery

## Abstract

**Introduction:**

Retroperitoneal duodenal perforation is a rare but severe complication of endoscopic procedures such as ERCP and ESD, often leading to significant morbidity and mortality when diagnosed late. Previous studies have focused on early diagnosis, but data on delayed diagnosis cases are limited. This study presents four cases of delayed-diagnosis retroperitoneal duodenal perforation and introduces a novel surgical management strategy.

**Presentation of case:**

We report four cases diagnosed more than one month after the initial endoscopic procedure, with CT scans revealing extensive retroperitoneal fluid collections and secondary colonic perforation in some cases. Surgical interventions included right hemicolectomy and proximal jejunostomy to facilitate duodenal exposure and clearance of infectious debris. Outcomes varied, with some patients achieving full recovery while others succumbed to complications.

**Discussion:**

The symptoms of retroperitoneal duodenal perforation are often subtle, leading to delayed diagnosis. Early surgical intervention is crucial if CT shows retroperitoneal fluid collections. Right hemicolectomy provides better duodenal exposure and facilitates effective debridement, improving outcomes.

**Conclusion:**

Early diagnosis and prompt surgical intervention, including right hemicolectomy, are essential for managing delayed retroperitoneal duodenal perforation. Further studies are needed to establish optimal management protocols for these complex cases.

## Highlights

• Retroperitoneal duodenal perforations are often subtle, leading to delayed diagnosis.
• Delayed retroperitoneal duodenal perforations cause extensive retroperitoneal fluid collections and colonic perforation.
• Early surgical intervention is recommended if CT shows retroperitoneal fluid collections.
• Right hemicolectomy provides full duodenal exposure for effective management of perforations.

## 1 Introduction

Retroperitoneal duodenal perforation is a severe complication of endoscopic treatment, particularly ERCP [1]. Stapfer classified these perforations into four types based on location, severity, and injury mechanism, and proposed corresponding treatment methods [2]. Previous case reports have primarily focused on early diagnosis and surgical timing from an endoscopist's perspective [3]. A delay of more than 24 h in the diagnosis and intervention of duodenal perforation can significantly increase both morbidity and mortality [4]. However, data on delayed diagnosis cases are limited, and the optimal surgical approach for such cases remains undetermined. This study presents four cases of delayed-diagnosis retroperitoneal duodenal perforation resulting from endoscopic treatment and introduces a novel surgical management strategy. This work has been published according to the PROCESS criteria [5].

## 2 Case presentation

This was a single retrospective study and case series of 4 patients with retroperitoneal duodenal perforation after endoscopic treatment at Beijing Friendship Hospital between September 2023 and May 2024. We obtained written informed consent from all patients.

Clinical characteristics of four cases were listed at Table 1. Three cases underwent ERCP + EST for choledocholithiasis, while one case had ESD for a large polyp opposite the ampulla. All patients experienced abdominal pain on the day of the procedure. Abdominal examination revealed right upper quadrant tenderness without rigidity or guarding. Three patients were diagnosed with pancreatitis due to elevated serum amylase without CT and were managed conservatively without strict fasting. Case 3 was diagnosed with Type I retroperitoneal duodenal perforation via CT on the procedure day.

**Table 1:** Clinical characteristics of four cases of retroperitoneal duodenal perforation after endoscopic treatment.

Case	Age/gender	Pre-perforation diagnosis	Endoscopic treatment	Time from endoscopic treatment to definitive diagnosis	Colonic perforation and timing	Time from definitive diagnosis to surgical intervention	Surgical approach	Perforation site identified during surgery	Perforation type according to stapler	Length hospital stay (days)	Outcome
Case 1	76/female	Choledocholithiasis	ERCP, EST, ENBD	1 month	11 days after definitive diagnosis, CT reported colonic perforation	16 days	Debridement + right hemicolectomy + proximal jejunostomy	Not identified	Type II	112	Survived
Case 2	78/male	Choledocholithiasis	ERCP + EST + ERPD + ENBD	22 days	27 days after definitive diagnosis, subsequent CT reported colonic perforation	28 days	Right hemicolectomy + proximal jejunostomy	Not identified	Type II	90	Death, pulmonary infection
Case 3	66/female	Large polyp opposite the ampulla of Vater	ESD of the large polyp	1 day	37 days after initial drainage surgery, colonic perforation occurred	19 days (first), 56 days (second)	1st, debridement and drainage; 2nd, right hemicolectomy + proximal jejunostomy	1st, not identified; 2nd, perforation site identified	Type I	73	Death, abdominal bleeding
Case 4	42/male	Choledocholithiasis	ERCP, EST	1.5 months	No colonic perforation	1 day	Right hemicolectomy + proximal jejunostomy	Not identified	Type II	24	Discharged

**Fig. 1:**
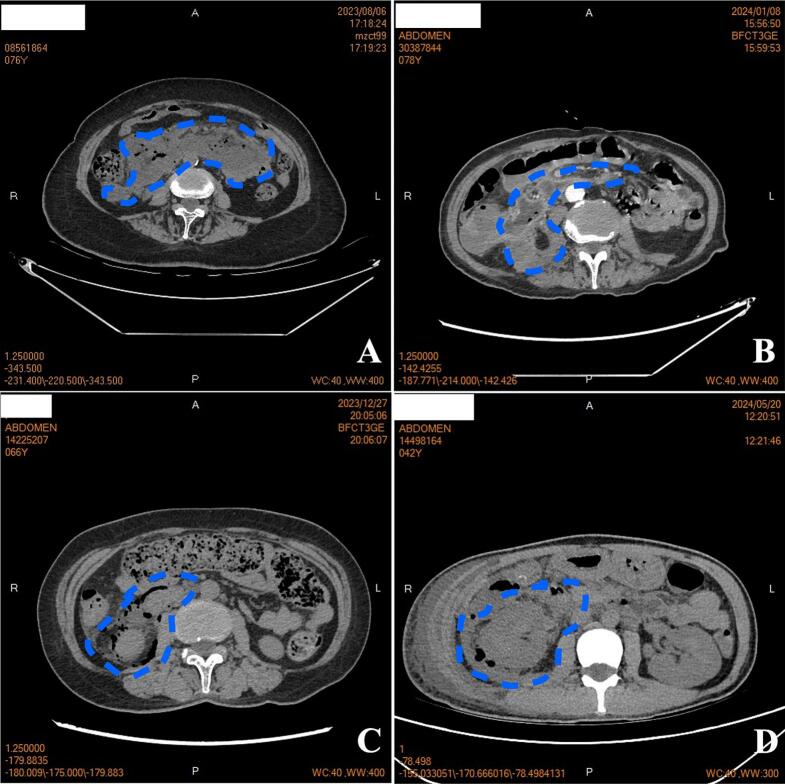
CT findings at initial diagnosis of retroperitoneal duodenal perforation. A, B, C, D were for case 1, 2, 3, 4 respectively. The area encircled by the blue line represents the exudate or gas accumulation in the retroperitoneal space.

The abdominal pain and fever initially were modest but progressively worsened. It took nearly a month to diagnose retroperitoneal duodenal perforation via CT in three cases. CT scans showed varying extents of fluid extravasation and pneumoretroperitoneum (Fig. 1). In Case 1, the extravasation extended from the duodenum's horizontal segment superiorly to the right iliac fossa inferiorly, with bilateral extension to the renal hila.

None of the patients underwent immediate surgery upon initial diagnosis of retroperitoneal perforation. Conservative management was initiated for varying durations (half a month to one month). Cases 1 and 2 had laparotomies on Day 16 and Day 28 post-diagnosis, respectively, due to colonic perforation at the hepatic flexure on follow-up CT. Case 3 had laparotomy on Day 19 post-diagnosis because the CT showed extensive exudative fluid in the right retroperitoneal space. Only Case 4 had surgery on the day of perforation diagnosis.

**Fig. 2:**
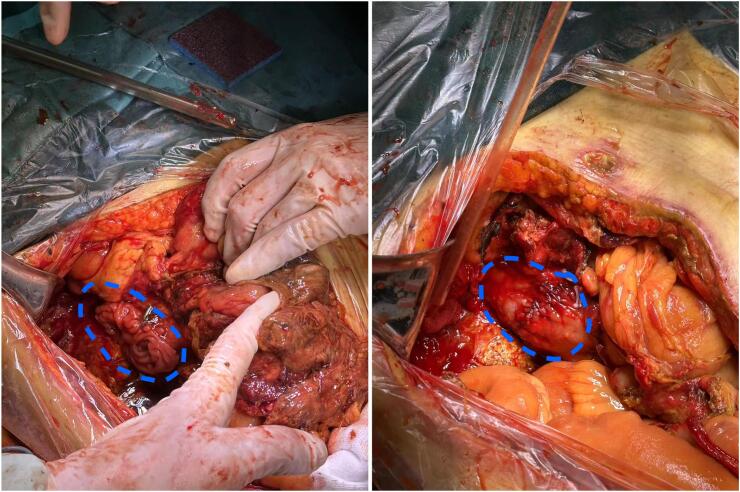
Identification and suture of duodenal perforation site following right hemicolectomy in Case 3. The blue circle marks the mucosa that is everted from the duodenal leak and the effect after suturing.

For Cases 1, 2, and 4, exploratory laparotomy revealed retroperitoneal abscesses. Right hemicolectomy with proximal jejunostomy was performed, and the duodenum was fully exposed. The perforation sites could not be identified, classifying them as Type II. For Case 3, first exploratory laparotomy was performed, with careful debridement of necrotic tissue. The surrounding tissues were severely inflamed, edematous, and friable. The duodenum could not be clearly exposed, and the perforation site was not identified. Drainage tubes were placed, but extravasation and necrotic tissue were difficult to drain. Subsequent CT scans showed increased retroperitoneal extravasation and colonic perforation, prompting a second surgery with right hemicolectomy (Video). After removing the ascending colon, the duodenum was fully exposed, and the 4-cm perforation in the descending portion was sutured (Fig. 2). The leakage and local infection were controlled.

Cases 1 and 4 recovered after 112 days and 24 days of hospital stay, respectively, with jejunostomy reversal. Case 2, with a history of COPD, succumbed to respiratory failure on post-laparotomy day 40. Case 3 died from abdominal cavity bleeding 15 days after the second surgery.

## 3 Discussion

### 3.1 The symptoms and diagnostic challenges of retroperitoneal duodenal perforation

The symptoms of retroperitoneal duodenal perforation are often subtle, with early fluid extravasation into the retroperitoneal space not typically causing significant abdominal pain or peritonitis [6]. This may lead clinicians to overlook the possibility of perforation and delayed CT scan which is the most suitable imaging modality. When patients present with abdominal pain or fever following endoscopic procedures such as ERCP or ESD, duodenal perforation should be considered alongside endoscopic-related pancreatitis. A CT scan or oral-contrast radiograph should be promptly performed to confirm or rule out perforation [7]. Failure to recognize duodenal perforation can lead to delayed and catastrophic outcomes [4].

In our study, several patients initially presumed to have endoscopic-related pancreatitis were later diagnosed with duodenal perforation. Some of these patients were managed according to the pancreatitis protocol and discharged after one week. Because the abdominal distention and pain gradually worsened, they sought medical attention again nearly a month later. Earlier diagnosis and treatment according to a duodenal perforation protocol, such as prompt CT scan and delaying the initiation of feeding, might have avoided surgical intervention.

### 3.2 The timing of surgical intervention

Type I perforations, usually large and located on the lateral wall of the duodenum, cause significant and persistent leakage of digestive juices into the retroperitoneal space. This fluid extravasation can extend to the extraperitoneal space of the lateral abdominal wall and the anterior bladder space, potentially eroding surrounding organs such as the colon. Immediate surgery is required for Type I perforations.

In our study, Case 3 underwent ESD for a large polyp on the duodenum's lateral wall and was diagnosed with retroperitoneal perforation but did not receive immediate surgery (delayed by 19 days). This delay led to extensive fluid extravasation and subsequent complications, highlighting the necessity of early surgical intervention for better outcomes.

Type II perforations, the most common type, occur near the ampulla of Vater. If diagnosed and managed promptly (within 24 h), most patients can avoid surgery [3] [8]., Serial CT scans are essential to monitor perforation progression. Increased fluid collection in the retroperitoneal space necessitates surgical intervention. Some cases showed only air collection in the retroperitoneal space on subsequent CT scans and do not require surgery [9].

In our study, three Type II perforations were diagnosed late, leading to colonic perforation due to excessive fluid extravasation. For such cases, surgical intervention should be considered if CT shows moderate fluid collection in the retroperitoneal space at initial diagnosis, as earlier intervention improves recovery.

### 3.3 Type of surgical intervention

For patients with perforations complicated by a large amount of retroperitoneal effusion, the treatment focus is on the drainage and clearance of the effusion. Therefore, the literature reports that surgical procedures include percutaneous catheter drainage or extraperitoneal incision [7,10]. However, these methods fail to address the leak site, often necessitating multiple surgeries and leaving complications like incision infections, sinus tracts, and gastroparesis. Surgical strategies to address the leak site include duodenal exclusion, gastrojejunostomy, and duodenostomy [7,10,11], which, while effective, disrupt the normal gastrointestinal pathway, impacting nutrient absorption.

Kocher and Cattell-Braasch maneuvers are classic surgical techniques for exposing the duodenum. However, in patients with delayed diagnosis, significant edema and inflammation of the retroperitoneal tissues and colon can limit the effectiveness of these maneuvers in mobilizing the right colon and mesentery to adequately expose the duodenum.

In our case series, three patients had perforations of the ascending colon (caused by corrosive effects of leaked digestive fluids) at the time of surgery. Therefore, we performed right hemicolectomy, which provided excellent exposure of the entire duodenum, facilitating the identification of the leak site and debridement of necrotic tissue. Right hemicolectomy is a well-established and relatively safe procedure for treating ascending colon cancer. The decision to perform an ileocolic anastomosis depends on the degree of edema and inflammation of the bowel ends, allowing for either primary anastomosis or initial ileostomy with subsequent closure.

In Case 3, a Type I perforation was diagnosed but surgical intervention was delayed. The initial surgery involved debridement and placement of drainage tubes but failed to stop the continuous leakage and subsequent colonic perforation, necessitating a second surgery. During the second surgery, right hemicolectomy fully exposed the duodenum, allowing identification and repair of a 4-cm perforation and effective control of retroperitoneal infection. There was significant edema of the ileal and colonic walls. Direct ileocolic anastomosis was not performed due to concerns about anastomotic leak, so the ileostomy with subsequent closure approach was chosen. Given the success of right hemicolectomy in the first three cases of our series, we performed this procedure immediately upon admission in the last delayed-diagnosis case, leading to a good recovery.

For Type I perforations, the perforation site can be readily identified following complete exposure of the duodenum, as demonstrated in Case 3. In contrast, for Type II perforations, identification of the perforation site remains challenging even with complete exposure, due to its peri-Vaterian location. In our case series, the perforation site could not be identified after right hemicolectomy in three cases. However, right hemicolectomy to fully expose the retroperitoneal abscess cavity is beneficial for the complete removal of debris. In our three Types II delayed-diagnosis patients, right hemicolectomy allowed for thorough debridement of necrotic and infected material, effectively controlling local infection. Two patients achieved full recovery, while one patient, due to the prolonged disease course, died from gastrointestinal bleeding caused by stress ulcers.

## 4 Conclusion

Delayed diagnosis of retroperitoneal duodenal perforation is challenging due to its subtle symptoms and potential for severe complications. Early surgical intervention is crucial, especially when CT scans reveal extensive retroperitoneal fluid collections. Right hemicolectomy emerges as a valuable surgical strategy, facilitating duodenal exposure and effective debridement. Further research is warranted to refine diagnostic and management protocols for delayed retroperitoneal duodenal perforation.

The following is the supplementary data related to this article.

Video Extensive retroperitoneal extravasation in Case 3. CT demonstrating fluid extension from the right retroperitoneal space to the anterior abdominal wall and inferiorly to the anterior interstitium of the bladder. Video

## CRediT authorship contribution statement

**Liu Yuan:** Writing- Original draft preparation, Methodology, Software. **Song Jianning**: Conceptualization, Writing - Review & Editing, Supervision. **Li Jun**: Resources, Data curation. **Sun Xiujing**: Resources, Data curation, Investigation. **Song Jiugang***:* Resources, Data curation**. Yao Hongwei**: Funding acquisition

## Consent

All patients detailed in this case series provided informed written consent.

## Ethical approval

This case series publication did not strictly meet the criteria of research. Although illustrative, it does not meet the Federal Policy for the Protection of Human Subjects definition of Research, which requires an investigation that contributes to generalizable knowledge about a disease or condition. Subsequently Ethics Committee deemed not necessary for a formal submission.

## Guarantor

Song Jianning (corresponding author)

## Funding

Fund program: Clinical Center for Colorectal Cancer of Capital Medical University (1192070313).

## Conflict of interest statement

All authors declare no conflict of interest in relation to the work described.
